# Cognitive and social cognitive function in patients with schizophrenia and affective disorder: effects of combining pharmacotherapy with cognitive remediation

**DOI:** 10.1192/j.eurpsy.2024.1587

**Published:** 2024-08-27

**Authors:** G. Sachs, A. Erfurth

**Affiliations:** ^1^ Medical University of Vienna; ^2^1st Department of Psychiatry and Psychotherapeutic Medicine, Klinik Hietzing, Vienna, Austria

## Abstract

**Introduction:**

In recent decades, there has been increasing interest in neurocognitive function, including non-social and social cognition. Cognitive impairment has a significant impact on functional outcome, especially in schizophrenic disorders, but also in affective and other psychiatric disorders.

**Objectives:**

It is our aim to present the assessment and measurement of cognitive dysfunction through adequate instruments and to evaluate the effects of combining pharmacotherapy and cognitive remediation.

**Methods:**

A review of the modern literature is undertaken and results of own investigations using the Screen for Cognitive Impairment in Psychiatry (SCIP, Sachs G *et al.* Schizophr Res Cogn. 2021 May 12;25:100197; Sachs G *et al.* Schizophr Res Cogn. 2022 Jun 6;29:100259) are presented and evaluated.

**Results:**

Our data show that it is possible to capture cognitive dysfunction in clinical practice.

**Conclusions:**

After a differentiated assessment of cognitive dysfunction, a specific combination of pharmacotherapy and cognitive remediation should be applied to patients with schizophrenia and affective disorders.

**Disclosure of Interest:**

None Declared

